# Socioeconomic position and use of hospital-based care towards the end of life: a mediation analysis using the English Longitudinal Study of Ageing

**DOI:** 10.1016/S2468-2667(20)30292-9

**Published:** 2021-02-08

**Authors:** Joanna M Davies, Matthew Maddocks, Kia-Chong Chua, Panayotes Demakakos, Katherine E Sleeman, Fliss E M Murtagh

**Affiliations:** a Cicely Saunders Institute of Palliative Care, Policy and Rehabilitation, Florence Nightingale Faculty of Nursing, Midwifery & Palliative Care, King's College London, London, UK; b Centre for Implementation Science, Health Service & Population Research, Institute of Psychiatry, Psychology & Neuroscience, King's College London, London, UK; c Department of Epidemiology & Public Health, University College London, London, UK; d Wolfson Palliative Care Research Centre, Hull York Medical School, University of Hull, Hull, UK

## Abstract

**Background:**

Many patients prefer to avoid hospital-based care towards the end of life, yet hospitalisation is common and more likely for people with low socioeconomic position. The reasons underlying this socioeconomic inequality are not well understood. This study investigated health, service access, and social support as potential mediating pathways between socioeconomic position and receipt of hospital-based care towards the end of life.

**Methods:**

For this observational cohort study, we included deceased participants from the nationally representative English Longitudinal Study of Ageing of people aged 50 years or older in England. We used a multiple mediation model with age-adjusted and gender-adjusted probit regression to estimate the direct effect of socioeconomic position (measured by wealth and education) on death in hospital and three or more hospital admissions in the last 2 years of life, and the indirect effects of socioeconomic position via three mediators: health and function, access to health-care services, and social support.

**Findings:**

737 participants were included (314 [42·6%] female, 423 [57·4%] male), with a median age at death of 78 years (IQR 71–85). For death in hospital, higher wealth had a direct negative effect (probit coefficient −0·16, 95% CI −0·25 to −0·06), which was not mediated by any of the pathways tested. For frequent hospital admissions, health and function mediated the effect of wealth (−0·04, −0·08 to −0·01), accounting for 34·6% of the total negative effect of higher wealth (−0·13, −0·23 to −0·02). Higher wealth was associated with better health and function (0·25, 0·18 to 0·33). Education was associated with the outcomes only indirectly via wealth.

**Interpretation:**

Our findings suggest that worse health and function could partly explain why people with lower wealth have more hospital admissions, highlighting the importance of socioeconomically driven health differences in explaining patterns of hospital use towards the end of life. The findings should raise awareness about the related risk factors of low wealth and worse health for patients approaching the end of life, and strengthen calls for resource allocation to be made on the basis of health need and socioeconomic profile.

**Funding:**

Dunhill Medical Trust Fellowship Grant (RTF74/0116).

## Introduction

Many patients prefer to remain at home towards the end of life,^1^ yet 90% have a hospital admission in the last year of life, and the number of admissions increases sharply during the last months of life.^2^ Reasons for end-of-life hospital admissions are complex; not all are inappropriate but many are considered avoidable.^3^ For this reason, death outside of hospital is used as an indicator of quality of end-of-life care.^4^ Despite policy initiatives in Europe and North America to support people to be cared for and die outside of hospital, end-of-life hospital admissions are common and hospital (*vs* home, inpatient hospice unit, or residential care or nursing home) remains the most common place of death in many countries.^5^

Characteristics that contribute to increased hospital use include individual factors (such as sex, ethnicity, and preferences), illness-related factors (such as functional status and diagnosis), and environmental factors (such as access to care at home).^6^, ^7^ In high-income countries, low socioeconomic position is consistently associated with dying in hospital (*vs* dying at home or in hospice) and with a higher number of hospital admissions in the last months of life.^8^

To understand why people with lower socioeconomic position experience more hospital-based care at the end of life, researchers need to investigate possible mediating pathways through which socioeconomic position influences care. Three potential pathways are through health and function, access to health-care services, and social support. People with lower socioeconomic position experience worse health, including a higher burden of disability and disease,^9^ and as a result might have higher need for hospital-based care at the end of life than those with higher socioeconomic position (health and function). People with lower socioeconomic position might access elective,^10^ primary,^11^ and social^12^ care services less, with access to transport being an important element of this,^13^ and therefore use hospital-based care more (access to healthcare services). The informal care and familial support systems that are essential for keeping people at home and out of hospital in the last months and years of life might be weaker among people with lower socioeconomic position (social support).^14^, ^15^

**Research in context**

**Evidence before this study**

There is consistent evidence that people with low socioeconomic position are more likely to experience hospital-based care towards the end of life. In our previous systematic review, we searched the MEDLINE, Embase, PsycINFO, CINAHL, and ASSIA databases without language restrictions from inception to Feb 1, 2019, for empirical observational studies from high-income countries reporting an association between any measure of socioeconomic position and end-of-life care outcomes, including death in hospital, and use of acute care. 112 studies of high-to-medium quality were included in the meta-analysis; quality was assessed using the Newcastle-Ottawa Quality Assessment Scale. Compared with people with the highest socioeconomic position, people with the lowest socioeconomic position were more likely to die in hospital than at home or in a hospice (pooled odds ratio from 31 studies: 1·30, 95% CI 1·23–1·38), and to receive acute hospital-based care in the last 3 months of life (pooled odds ratio from eight studies: 1·16, 1·08–1·25). We found no studies that investigated mediating pathways to explain why people with lower socioeconomic position experience more hospital-based care towards the end of life.

**Added value of this study**

To our knowledge, this is the first study to empirically test potential mediating pathways between socioeconomic position and use of hospital-based care towards the end of life. We used data from deceased participants of the English Longitudinal Study of Ageing, a representative sample of people in England aged 50 years or older. We analysed the direct effect of wealth and education on two outcomes—death in hospital and three or more hospital admissions in the last 2 years of life—and the indirect effects via the three mediators: health and function, access to health-care services, and social support.

People with lower wealth were more likely to die in hospital and had more hospital admissions compared to people with higher wealth. Worse health and function accounted for a third of the effect of wealth on hospital admissions. None of the pathways tested mediated the relationship between wealth and death in hospital. Education was associated with the outcomes only indirectly via wealth, reflecting that asset accumulation across the life-course is more relevant to end-of-life care than early-life socioeconomic position.

**Implications of all the available evidence**

In this representative sample, worse health partly explains why people with lower wealth had more hospital admissions in the last years of life. This finding challenges behavioural explanations for socioeconomic patterning in the use of hospital care towards the end of life, instead highlighting the importance of health-related need in driving inequality. These results suggest that health should not be treated simply as a confounder of socioeconomic position but rather as a factor on the pathway between socioeconomic position and hospital admissions. This work should heighten awareness among health-care professionals and commissioners about the related risk factors of low wealth and worse health for patients approaching the end of life. The precise mechanism through which wealth influences death in hospital remains unexplained. Efforts to investigate how asset ownership and income drives this relationship should continue.

Nationally representative longitudinal cohort studies offer an opportunity to study the socioeconomic determinants of end-of-life care in detail. The aim of this study was to investigate potential pathways between socioeconomic position and receipt of hospital-based care towards the end of life. The objective was to estimate the relative contribution of education, wealth, and three potential mediators—health and function, access to health-care services, and social support—on death in hospital and frequent hospital admissions in the last 2 years of life.

## Methods

### Study design and participants

For this observational cohort study, we used longitudinal data from the English Longitudinal Study of Ageing (ELSA). ELSA is a nationally representative longitudinal study collecting interview and self-completion questionnaire data approximately every 2 years on the health and social situation of between 8000 and 12 000 people aged 50 years or older living in England, beginning with wave 1 in 2002.^16^ For deceased ELSA participants, an end-of-life interview is carried out in person with a close relative, friend, or carer to obtain information on the last year of life (see appendix p 2 for more details).

We included all deceased ELSA participants with at least one wave of data collected before their death, and an end-of-life interview completed by a proxy. We excluded participants with an admission to a residential care or nursing home (care home) in the last 2 years of life. Admission to a care home might moderate the relationship between socioeconomic position and hospital-based care by reducing admissions for all residents.^17^ However, the ELSA sample of care home residents is too small to investigate these effects, thus care home residents were excluded from the main analysis. Participants recorded as having died in an ambulance or locations other than a hospital, home, hospice, or care home were excluded because of the difficulty classifying these locations in a binary outcome.

All participants gave written informed consent at each wave. Ethical approval for ELSA was granted from the NHS Research Ethics Committee (London Multicentre Research Ethics Committee, MREC/01/2/91). No additional ethical approval was required for this secondary analysis study.

### Study variables and preliminary analysis

For each participant, we included data on two socioeconomic position exposures (wealth and education, measured at each participant's first wave), three latent mediators (measured at each participant's final wave), and two outcomes (place of death and hospital admissions, measured at the end-of-life proxy interview; table 1). We also included age at death and gender as confounders influencing each of the mediators and outcomes.

**Table 1:** Summary of variables

	**Details or comparator**
**Socioeconomic position exposures (measured at baseline wave)**	
Highest educational qualification (self-reported)	Five hierarchical categories: (1) no formal qualifications, (2) lower secondary (GCE, O Level, or equivalent), (3) higher secondary (A Level or equivalent), (4) higher education (below degree level), and (5) degree
Wealth (self-reported)	Deciles (1=lowest) of total net non-pension household wealth: a sum of savings, investments, physical wealth, and housing wealth after financial debt and mortgage debt has been subtracted, reflecting accumulation of assets over the life course^18^
Mediators (measured at final wave)*	
Health and function (self-reported and nurse collected)	Validated Latent Index of Somatic Health including chronic illness (physical and mental), mobility, general health, and nurse-collected measures (hand grip strength, forced vital capacity, and chair rise time)^19^
Access to health-care services (self-reported)	Latent measure of ease of access to services (general practice, dentist, optician, and hospital), unmet social care need, and transport deprivation
Social support (self-reported)	Latent measure of quality of relationships with children, family, and friends
**Outcomes (measured at end-of-life proxy interview)**	
Death in hospital (proxy reported)	Compared with death at home (including own home, another person's home, and sheltered housing [not including care homes]) or in an inpatient hospice unit†
At least three hospital admissions in the last 2 years of life (proxy reported)	Compared with up to two hospital admissions (including the terminal admission if the person died in hospital)‡
**Covariates**	
Age at death (self-reported)	Also used as a moderator
Gender (self-reported)	..

GCE=General Certificate of Education.

* For latent mediators, high scores were optimal.

† In the UK, hospice is almost always a separate setting to hospital.

‡ The cutoff for number of hospital admissions reflects the data distribution.

We analysed the distribution of all variables using percentages, means, and medians. Outcomes and exposures were described separately for participants younger than 80 years at death and those aged 80 years or older.

We modelled each of the mediators as continuous latent factors. Details of the items used in the latent mediators are provided in the appendix (pp 5–7). Variables representing factors were selected based on a-priori hypotheses and combined using confirmatory factor analysis. Latent factor scores were extracted and used in subsequent models.

To understand the relationships between variables before testing the full structural model, we analysed paths between outcomes, mediators, and exposures in separate regression models, controlling for age and gender, and analysed single mediator models. Statistical significance was set a priori at p<0·05 with no adjustment for multiplicity.

### Full structural model

All hypothesised mediators were included in the final model simultaneously. Multiple mediation is more “convenient, precise and parsimonious” than using multiple single mediation models, and might help to reduce parameter bias due to omitted variables.^20^

Probit regression with a weighted least-squares estimator (see appendix p 4 for technical information) was used to estimate the direct effect of socioeconomic position exposures on the outcomes, and the indirect effects of the exposures on the outcomes via the mediators. We described the extent of mediation as the proportion of the total effect of an exposure mediated by a specific indirect effect. We treated socioeconomic position sequentially, with education (usually set in early adulthood) antecedent to wealth (a measure of assets accumulated over the life-course^18^). Health and function was allowed to affect access to health-care services because health need is a prerequisite of accessing services. We specified a correlational, rather than a directional, relationship between social support and access to health-care services because of a lack of evidence on how quality of relationships might influence access. We report the residual covariance between the two outcome variables.

Results are presented as standardised coefficients from probit regression. We interpreted the probit coefficients in terms of direction, magnitude, and statistical significance. To aid interpretation of the effects, we translated some coefficients to probabilities. Probabilities are for a man with average age, health and function, access to health-care services, and social support. Model fit was assessed using root mean square error of approximation (RMSEA; values of ≤0·06 representing good fit and ≤0·08 representing adequate fit), comparative fit index (CFI; ≥0·95 representing good fit and ≥0·90 representing adequate fit), and Tucker-Lewis index (TLI; ≥0·95 representing good fit and ≥0·90 representing adequate fit).^21^

The proportion of missing data was low (<5%) for all variables apart from the latent social support variable (26·1% missing). To address this, in the full structural model, missing data were imputed using all variables in the model and 30 sets, with estimates and model fit indices averaged using Rubin's rules (appendix p 4).

### Moderation by age

Previous studies have shown that the influence of socioeconomic position on health might weaken with increasing age.^22^ We examined this potential moderation effect by plotting the age-moderated effect of socioeconomic position exposures on health and function and on the outcomes, to see if the magnitude of the direct effects weakened with increasing age. Age was centred to aid interpretation. The literature offered no substantive justification for examining other potential moderation effects (eg, gender).

### Sensitivity analysis

We did three sensitivity analyses. First, we repeated the final analysis for a sample including participants with a care home admission to evaluate how exclusion of this subgroup from the main sample affected results. Second, bootstrapped model estimates using 5000 draws and based on non-imputed data were obtained and compared with the final model estimates based on the data from multiple imputation.

Diagnosis (and cause of death) is socially patterned and might influence end-of-life care—for example, hospital death is less likely for people dying from cancer compared with non-cancer conditions.^23^ Our model treats disease as a potential mediator on the pathway from socioeconomic position to care and therefore does not additionally control for specific diagnoses. In a third sensitivity analysis, to investigate potential confounding of the exposure–outcome relationships by diagnosis, we adjusted effects on the outcomes for cancer as a cause of death and diagnosis of depressive symptoms.

Data preparation was carried out in Stata (version 13). Analysis was carried out in Mplus (version 8.1).

### Role of the funding source

The funder of the study had no role in study design, data collection, data analysis, data interpretation, or writing of the report.

## Results

**Figure 1:**
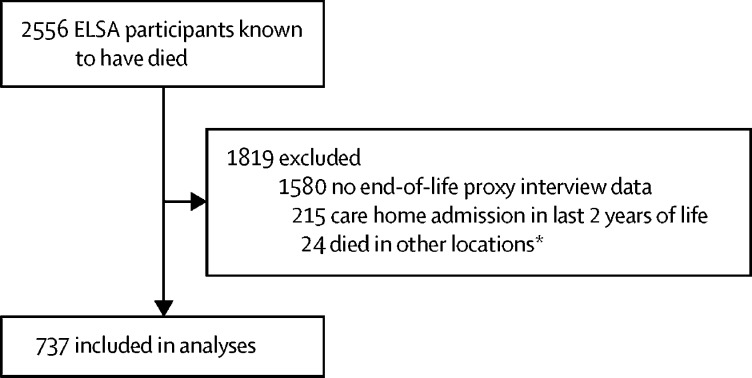
Inclusion and exclusion of participants ELSA=English Longitudinal Study of Ageing. *Locations other than a hospital, home, hospice, or care home.

After exclusions, 737 ELSA participants were included in the final sample (figure 1); 423 (57·4%) were men, 412 (55·9%) were younger than 80 years old when they died, and 718 (97·4%) identified as white (table 2). At their final wave, 542 (73·5%) had one or more chronic illnesses and 434 (58·9%) had one or more functional limitation (appendix p 5).

**Table 2:** Characteristics for 737 deceased ELSA participants

		**All participants (n=737)**	**Participants who died aged <80 years (n=412)**	**Participants who died aged ≥80 years (n=325)**
Median age at death, years		78 (71–85)	..	..
Gender				
	Female	314 (42·6%)	168 (40·8%)	146 (44·9%)
	Male	423 (57·4%)	244 (59·2%)	179 (55·1%)
Surviving spouse or partner at time of death		452 (61·3%)	298 (72·3%)	154 (47·4%)
Cause of death				
	Cancer	251 (34·1%)	186 (45·1%)	65 (20·0%)
	Cardiovascular disease	215 (29·2%)	97 (23·5%)	118 (36·3%)
	Respiratory disease	92 (12·5%)	43 (10·4%)	49 (15·1%)
	Other	84 (11·4%)	43 (10·4%)	41 (12·6%)
	Missing	95 (12·9%)	43 (10·4%)	52 (16·0%)
Place of death				
	Home	219 (29·7%)	140 (34·0%)	79 (24·3%)
	Hospital	449 (60·9%)	217 (52·7%)	232 (71·4%)
	Hospice	69 (9·4%)	55 (13·3%)	14 (4·3%)
Number of hospital admissions in the last 2 years of life				
	≥3	187 (25·4%)	114 (27·7%)	73 (22·5%)
	<3	539 (73·1%)	293 (71·1%)	246 (75·7%)
	Missing	11 (1·5%)	5 (1·2%)	6 (1·8%)
Wealth quintile at baseline*				
	1 (lowest)	195 (26·5%)	102 (24·8%)	93 (28·6%)
	2	150 (20·4%)	86 (20·9%)	64 (19·7%)
	3	135 (18·3%)	77 (18·7%)	58 (17·8%)
	4	127 (17·2%)	70 (17·0%)	57 (17·5%)
	5 (highest)	125 (17·0%)	75 (18·2%)	50 (15·4%)
	Missing	5 (0·7%)	2 (0·5%)	3 (0·9%)
Education at baseline				
	No formal qualification	447 (60·7%)	226 (54·9%)	221 (68·0%)
	Lower secondary	129 (17·5%)	82 (19·9%)	47 (14·5%)
	Higher secondary	38 (5·2%)	23 (5·6%)	15 (4·6%)
	Higher education (below degree)	52 (7·1%)	37 (9·0%)	15 (4·6%)
	Degree	70 (9·5%)	44 (10·7%)	26 (8·0%)
	Missing	1 (0·1%)	0	1 (0·3%)

Data are n (%) or median (IQR). ELSA=English Longitudinal Study of Ageing.

* In deciles for main analysis.

Participants contributed to a median of two waves (IQR 1–3) before death. Baseline was a median of 27·4 months (0·0–50·7) before the final wave. The final wave was a median of 15·2 months (8·1–21·3) before death and the end-of-life proxy interview a median of 20·3 months (12·2–32·0) after death. Deaths occurred between 2002 and 2012.

The three measurement models had good fit to the data (appendix p 8). For the final structural model, values of the χ^2^ statistic (χ^2^[3] = 5·946; SD 2·208), RMSEA (0·033; SD 0·015), and CFI (0·992; SD 0·006) indicated good fit, and the TLI (0·909; SD 0·068) indicated adequate fit.

**Figure 2:**
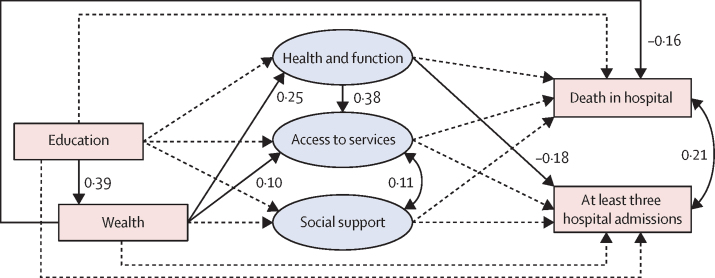
Standardised probit coefficients for the direct effects for the final full structural model Single-headed arrows indicate regression paths, double-headed arrows indicate covariances, ovals represent latent variables, and rectangles represent measured variables. Coefficients are shown for statistically significant paths, whereas paths with dashed lines were not significant.

In the preliminary analysis (appendix p 9) and the final model (figure 2; table 3), higher wealth was associated with better health and function and better access to health-care services, and negatively associated with death in hospital. None of the pathways tested mediated the effect of wealth on hospital death. In the final model, the predicted probability of death in hospital for a man in the lowest decile of wealth was 69·9%, compared with 50·8% in the highest decile of wealth.

**Table 3:** Standardised probit coefficients for the direct and indirect effects for the final full structural model

		**Wealth**	**Health and function**	**Access to health-care services**	**Social support**	**Death in hospital**	**At least three hospital admissions**
**Covariates**							
Age		..	−0·04 (−0·12 to 0·03)	−0·23 (−0·29 to −0·17)*	0·12 (0·03 to 0·20)*	0·26 (0·17 to 0·35)*	−0·14 (−0·24 to −0·04)*
Female gender		..	−0·11 (−0·26 to 0·03)	0·10 (−0·04 to 0·23)	0·27 (0·10 to 0·44)*	−0·01 (−0·19 to 0·18)	−0·16 (−0·36 to 0·05)
**Mediators**							
Health and function (direct effect)		..	..	0·38 (0·32 to 0·45)*	**..**	−0·04 (−0·14 to 0·05)	−0·18 (−0·29 to −0·07)*
	Indirect effect via access to health-care services	..	..	..	..	0·02 (−0·02 to 0·06)	0·00 (−0·04 to 0·05)
Access to health-care services (direct effect)		..	..	..	..	0·06 (−0·04 to 0·16)	0·00 (−0·10 to 0·12)
Social support (direct effect)		..	..	..	..	0·04 (−0·07 to 0·14)	−0·05 (−0·16 to 0·06)
**Wealth**							
Direct effect		..	0·25 (0·18 to 0·33)*	0·10 (0·02 to 0·17)*	0·08 (−0·02 to 0·17)	−0·16 (−0·25 to −0·06)*	−0·08 (−0·19 to 0·03)
Total indirect effects		..	..	..	..	0·01 (−0·02 to 0·03)	−0·05 (−0·08 to −0·01)*
	Via health and function	..	..	0·10 (0·06 to 0·13)*	**..**	−0·01 (−0·04 to 0·01)	−0·04 (−0·08 to −0·01)*
	Via access to health-care services	..	..	..	..	0·01 (−0·01 to 0·02)	0·00 (−0·01 to 0·01)
	Via social support	..	..	..	..	0·00 (−0·01 to 0·01)	−0·00 (−0·02 to 0·01)
	Via health and function, and access to health-care services	..	..	..	..	0·01 (−0·00 to 0·02)	0·00 (−0·01 to 0·01)
Total effect		..	..	..	..	−0·15 (−0·25 to −0·06)*	−0·13 (−0·23 to −0·02)*
**Highest educational qualification**							
Direct effect		0·39 (0·32 to 0·46)*	0·00 (−0·08 to 0·08)	0·03 (−0·05 to 0·11)	−0·00 (−0·09 to 0·09)	−0·09 (−0·18 to 0·01)	0·01 (−0·10 to 0·12)
Total indirect effects		..	..	..	..	−0·06 (−0·10 to −0·02)*	−0·05 (−0·09 to −0·01)*
	Via health and function	..	..	0·00 (−0·03 to 0·03)	..	−0·00 (−0·00 to 0·00)	0·00 (−0·01 to 0·01)
	Via access to health-care services	..	..	..	..	0·00 (−0·00 to 0·01)	0·00 (−0·00 to 0·00)
	Via social support	..	..	..	..	0·00 (−0·00 to 0·00)	0·00 (−0·01 to 0·01)
	Via wealth	..	0·10 (0·06 to 0·13)*	0·04 (0·01 to 0·07)*	0·03 (−0·01 to 0·07)	−0·06 (−0·10 to −0·02)*	−0·03 (−0·07 to 0·01)
	Via health and function, and access to health-care services	..	..	..	..	0·00 (−0·00 to 0·00)	0·00 (0·00 to 0·00)
Total effect		..	..	..	..	−0·14 (−0·23 to −0·05)*	−0·04 (−0·14 to 0·06)
**Covariances**							
Social support		..	..	0·11 (0·03 to 0·19)*	**..**	**..**	**..**
Death in hospital		..	..	**..**	**..**	**..**	0·21 (0·08 to 0·34)*

Model includes data from 737 participants.

* p value <0·05.

In the preliminary analysis (appendix p 9), higher wealth was negatively associated with hospital admissions; the probability of having three or more hospital admissions was 34·5% for a man in the lowest decile of wealth and 29·5% in the highest decile of wealth. In the final model (table 3), health and function mediated the effect of wealth on admissions, accounting for 34·6% of the total effect of wealth on admissions, and the direct effect of wealth on admissions was no longer significant.

In the preliminary analysis (controlling for wealth) and in the final model, we found no significant direct effect for education on the outcomes or mediators. In the final model, higher education had a strong positive effect on wealth, and indirect effects via wealth on health and function, access to health-care services, and death in hospital.

Better health and function was negatively associated with frequent hospital admissions but had no significant association with death in hospital in the preliminary analysis (appendix p 9) or in the final model (table 3). Better health and function was associated with better access to health-care services in the final model. Access to health-care services was not associated with the outcomes in the preliminary analysis or final model. Social support was not associated with the exposures or outcomes in the preliminary analysis or in the final model. Social support and access to health-care services were correlated in the final model (table 3).

**Figure 3:**
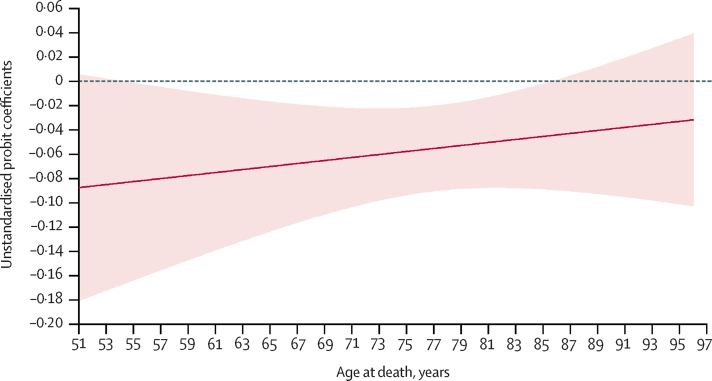
Direct effect of wealth on death in hospital (*vs* home or hospice) Figure plots the direct effect (with 95% CIs) of wealth on death in hospital, moderated by age.

The negative effect of increased wealth on likelihood of death in hospital was weaker at older ages (figure 3). The effect was not significant for people who died aged 85 years or older or for the very youngest in the sample (figure 3). The probability of death in hospital for the oldest participants was 70·5% for the most deprived and 60·0% for the least deprived, whereas for the youngest participants, the probability of death in hospital was 68·8% for the most deprived and 38·2% for the least deprived. The positive direct effect of wealth on health and function also diminished as age increased (appendix p 10).

In our sensitivity analyses, the results from the bootstrapped model estimates were largely consistent with the final model estimates (appendix p 11). Effects in the sample that included care home residents (n=950) were similar to the those in the main sample, although the effect of wealth on hospital death appears attenuated when care home residents are included (appendix p 12). When controlling (separately) for cancer diagnosis and depressive symptoms, the effect of wealth on hospital death was attenuated but remained significant (appendix p 14).

## Discussion

In this nationally representative sample, lower wealth was associated with an increased probability of having three or more hospital admissions in the last 2 years of life and an increased probability of death in hospital (*vs* death at home or in hospice). Health and function mediated the relationship between wealth and hospital admissions, accounting for a third of the total effect of wealth. This suggests that people with lower wealth have more hospital admissions in part because they have worse health. None of the pathways tested, including health and function, mediated the relationship between wealth and hospital death. Education was only associated with the outcomes indirectly via wealth, reflecting that asset accumulation across the life course is likely to be more relevant to end-of-life care than early life socioeconomic position.

In our preliminary analysis, lower wealth was associated with both lower access to health-care services and higher hospital admissions. A recent population-based study reported similar findings; the authors suggested that these separate associations could represent a substitution effect, with people with lower socioeconomic position substituting emergency care for elective care.^10^ Our structural model tested this potential pathway but found that lower access to health-care services among people with lower wealth did not explain their higher hospital admissions. Instead, we found that worse health and function partly explained the effect of lower wealth on hospital admissions, challenging behavioural explanations for higher use of hospital care among people with lower socioeconomic position.

A recent simulation study showed that sample size requirements for structural equation models range from 30 to 460 and depend on a multitude of factors.^24^ Missing data and small effect sizes, inherent in observational data and present in this study, can limit statistical power.^24^ Results from our study should be interpreted with caution and, where possible, tested in alternative data sources. Strengths of this study are the relatively large sample size (n=737), application of multiple imputation methods, and use of the ELSA data, a unique resource for studying the relationship between socioeconomic position and hospital-based end-of-life care. A further strength is that we tested multiple competing hypotheses; however, our model does not rule out the possibility of omitted mediators and confounders that might bias our results. In this secondary analysis, the choice and timing of measures was constrained by the design of the primary survey, leaving some aspects potentially relevant to end-of-life care unrecorded (eg, social support and frequency of contacts closer to death, use of advance care planning, and access to community-based end-of-life care services).^6^, ^8^

Social patterning in cause of death might explain some of the effect of wealth on death in hospital. For example, in our sample (appendix p 13) and in other studies, dying from cancer is associated with higher socioeconomic position and lower likelihood of death in hospital.^23^ Our sensitivity analysis adjusted the final model for cancer as a cause of death and found that the direct effect of wealth on hospital death was attenuated but remained significant. Similar attenuating effects were found after adjusting for diagnosis of self-reported depressive symptoms. By not accounting for specific diagnoses or causes of death, our model might overestimate the magnitude of the direct effect of wealth on hospital death, although the relative contribution of the direct and indirect pathways would not be expected to change.

In this study, worse health and function predicted more frequent admissions but did not predict death in hospital. In some conditions, worse health might protect against dying in hospital by making terminal prognosis more predictable and hence planning for home death more possible.^25^ Investigating variation in the mediating role of the severity of disease and disability in different conditions could help to unpick this potentially bidirectional effect. Our analysis finds that after adjusting for social patterning in health and function, the direct effect of wealth on death in hospital remains. Further work to identify the particular aspects of income or material asset ownership that drives the relationship between wealth and hospital death is needed to inform strategies for reducing death in hospital for the most socioeconomically deprived people. Geographical inequality in the provision of hospice, care home, and hospital services are potentially modifiable factors in the relationship between socioeconomic position and end-of-life care that are also important to understand.^26^

The majority of our sample had no educational qualifications, reflecting that many older people left school at the minimum age without formal qualifications.^27^ Wealth deciles were better than education for differentiating between participants with lower socioeconomic position, which might partly explain the stronger effect wealth has on the outcomes. Studies designed to compare different socioeconomic position measures have found that wealth is a stronger predictor of death than early-life measures of socioeconomic position such as education and occupational class.^18^ An explanation for this is that wealth is closer chronologically to later-life health and reflects both current and accumulated socioeconomic position. Studies designed to measure other potentially modifiable factors on the pathway from education to care such as health literacy should be used to further investigate the impact of patient education on end-of-life care.

Recall bias is a possibility in this study given the retrospective nature of our proxy-reported outcomes. However, the sample distribution of the outcomes was a good reflection of patterns of hospital admissions and place of death in the wider population, suggesting small bias in the ELSA data (appendix p 3). Our treatment of place of death assumes that hospital death is a worse outcome than death at home or in hospice. It is important to acknowledge that place of death is an imperfect indicator of quality of care, as hospital might be the most appropriate or preferred place of death for some people. The measure of hospital admissions did not delineate between emergency and elective admissions and was based on the last 2 years of life, which might be a longer time period than would normally be considered as end of life.

The sample was subject to selection effects, biased towards including younger, wealthier men who had a living proxy to complete the end-of-life survey. This might weaken the effects of low wealth on the outcomes, particularly for women in the sample. Our main analysis excluded the important subgroup of people who move to a care home towards the end of life. Attenuation of wealth effects in our sensitivity analysis including care home residents supports the hypothesis that care home admission might moderate the relationship between socioeconomic position and hospital-based care.^17^ This warrants further study in a sample more representative of the population of care home residents.

Hospital admissions towards the end of life are common; not all admissions are inappropriate but many are considered avoidable.^3^, ^4^ There is consistent evidence that people with lower socioeconomic position are more likely to experience hospital-based care at the end of life.^8^ To our knowledge, this is the first study to attempt to empirically investigate factors mediating the relationship between socioeconomic position and receipt of hospital-based care towards the end of life. In this study, people with lower wealth experienced more hospital admissions in the last 2 years of life in part because they had worse health and function than wealthier people. A tendency to seek behavioural explanations for higher use of hospital care among more deprived groups^28^ might overlook that this relationship is driven by greater health needs. The relationship between lower wealth and increased probability of death in hospital was not explained by health and function. Efforts to understand how income and asset ownership might drive this relationship, and to test other potential mediators, including access to community-based end-of-life care services, should continue.

More socioeconomically deprived people experience a disproportionate burden of disability and disease.^9^ This study concludes that socioeconomically driven health differences might explain patterns of hospital admissions towards the end of life. Acknowledging that the greater burden of disease experienced by those with lower socioeconomic position also drives hospital admissions in the last years of life is important for policy and practice. The findings from this study strengthen calls for resource allocation formulae to ensure that funding of services is made on the basis of health need and socioeconomic profile,^9^ and should raise awareness among professionals providing end-of-life care about the related risk factors of low socioeconomic position and poor health. The methodological implications of this work are that studies investigating the role of socioeconomic position on hospital admissions should account for the mediating influence of health, rather than simply controlling for health as a confounder.

## Data sharing

Data used in this study are freely available from the UK Data Service. The analytical code is available on GitHub.
