# Supplementary material for: Socioeconomic position and use of hospital-based care towards the end of life: a mediation analysis using the English Longitudinal Study of Ageing
Source: Lancet Public Health. 2021 Feb 8;6(3):e155–63. doi: 10.1016/S2468-2667(20)30292-9 (PMC7910274; doi:10.1016/S2468-2667(20)30292-9)
Supplement: Supplementary appendix [file mmc1.pdf]

THE LANCET
Public Health

Supplementary appendix

This appendix formed part of the original submission and has been peer reviewed. We post it as supplied by the authors.

Supplement to: Davies JM, Maddocks M, Chua K-C, Demakakos P, Sleeman KE, Murtagh FEM. Socioeconomic position and use of hospital-based care towards the end of life: a mediation analysis using the English Longitudinal Study of Ageing. *Lancet Public Health* 2021; published online Feb 8. http://dx.doi.org/10.1016/S2468-2667(20)30292-9.

Appendix

1: ELSA end-of-life interview ... 2

Fieldwork dates: ... 2

Comparison of the sample with an end-of-life proxy interview, with the wider deceased ELSA cohort ... 2

2: Technical information on Mplus analysis ... 4

Standardisation procedure ... 4

Confirmatory Factor Analysis (CFA) ... 4

Preliminary analysis ... 4

Full structural model ... 4

Missing data ... 4

Multiple imputation (MI) ... 4

3: Descriptive analysis of the latent variables ... 5

Health and function ... 5

Access to healthcare services ... 6

Social support ... 7

Summary of latent variable measurement models ... 8

Standardised factor loadings ... 8

4: Preliminary analysis ... 9

5: Interaction effect showing the direct effect (and 95% CI) of wealth on health and function moderated by age ... 10

6: Standardised probit coefficients with bias corrected bootstrap confidence intervals for the direct (DIR) and indirect (IND) effects for the final structural model (non-imputed data), n=736 ... 11

7: Standardised probit coefficients for the direct (DIR) and indirect (IND) effects for the full structural model, n=950 including participants with an admission to a care home in the last 2 years of life ... 12

8: Sensitivity analysis: cause of death and depressive symptoms as potential confounders ... 13

8a Distribution of exposures, mediators and outcomes by cause of death* ... 13

8b Distribution of exposures, mediators and outcomes by self-reported depressive symptoms* ... 14

8c Direct (DIR) effect of wealth on the outcomes after adjusting for cancer cause of death (versus non-cancer cause of death), and diagnosis of depressive symptoms (versus absence of depressive symptoms) (n=737) ... 14

References ... 15

1: ELSA end-of-life interview

The end-of-life interview was introduced at wave 2 of ELSA, and carried out at wave 3, 4 and 6 (but not wave 5). It consisted of a face-to-face interview with a close friend, relative or carer of the deceased ELSA member and lasted approximately 30 minutes. The interview focused on the health and care needs of the deceased in the year proceeding their death and financial questions regarding inheritance and funeral expenses.

The wider ELSA collects data from 'core' sample members and the cohabiting partners of core sample members. Only 'core' ELSA members who died were eligible for a proxy to be approached for an end-of-life interview. Sample members were identified as having died from NHS mortality data linked to the ELSA (last updated following wave 5), or from individual updates received during fieldwork or between waves. End-of-life interviewees were prioritised in the following order: i) informant identified by interviewer in the field (when the interviewer was informed of the member's death), ii) another ELSA member (partner member) in the household, iii) previously identified proxy (when the member had previously provided contact details for a nominated proxy), iv) no informant identified/case no longer eligible for end-of-life interview. Information on the type of proxy interviewee is not available in the dataset. Limited information on the end-of-life interviewees is available and is provided in the table below.

End-of-life interviews were not sought if the death was too recent – less than 6 months prior to fieldwork. These deaths were then eligible for an end-of-life interview at subsequent waves. Deaths occurring more than two waves previously were no longer eligible.

Further details for the ELSA study, including procedural information about the sampling and fieldwork, data dictionaries, and study documentation including consent forms, interview guides and questionnaires are available from: https://www.elsa-project.ac.uk/study-documentation.

Characteristics for the end-of-life proxy interviewees

	N=976
**Gender:**	
Male	314 (32.2%)
Female	662 (67.8%)
**Relationship of respondent to deceased:**	
Husband/wife/partner	456 (46.7%)
Son/daughter/grandchild (natural or in-law)	382 (39.1%)
Parent/parent-in-law	15 (1.5%)
Brother/sister (natural or in-law)	34 (3.5%)
Other relative	38 (3.9%)
Other non-relative	51 (5.2%)

Fieldwork dates:

Wave 2: June 2004-July 2005
Wave 3: May 2006-August 2007
Wave 4: May 2008-July 2009
Wave 5 (no end-of-life interviews): June 2010-July 2011
Wave 6: May 2012-June 2013

Comparison of the sample with an end-of-life proxy interview, with the wider deceased ELSA cohort

2,556 core ELSA participants are known to have died, of these 976 have a complete end-of-life proxy interview. Compared to the deceased cohort with no end-of-life proxy data, the end-of-life proxy subset has fewer women (50.4% and 45.8%, respectively, X^2 (1) = 5.0672, p=0.024), fewer aged 80 years and above when they died (58.0% and 51.1%, respectively, X^2 (1) = 11.4474, p=0.001), and are wealthier with fewer in the most deprived quintile for wealth at baseline (33.1% and 27.7%, respectively, X^2 (4) = 13.9343, p=0.008). These demographic differences reflect that deceased ELSA participants who were younger and male were more likely to have a living proxy to complete the interview than the older and female decedents.

Distribution of place of death and hospital admissions in the deceased ELSA participants and in the wider population for England

The following tables present the distribution of place of death and hospital admissions for deceased ELSA participants with end-of-life proxy data (n=976), and for the wider population in England using published sources referenced below.

Distribution (n (%)) of place of death, comparing deceased ELSA participants to all deaths in England between 2001-2010[1]

	Deceased ELSA participants (n=975*)	All deaths in England, 2001-2010 (n=4,567,704)
Hospital	527 (54.0%)	2617294 (57.3%)
Home	228 (23.4%)	867864 (19.0%)
Hospice	72 (7.4%)	232953 (5.1%)
Care home	122 (12.5%)	785645 (17.2%)
Other/elsewhere	26 (2.7%)	63948 (1.4%)

*excluding 1 participant with missing place of death information

X^2 (4) = 45.2545 (p=3.52)

Distribution of number of hospital admissions in the last 2 years of life for deceased ELSA participants, and hospital admissions in the last 12 months of life for all people who died in England between April 2009-March 2012[2]

Notes on the comparability of this data:

In the Bardsley *et al* study the sample excluded 9.8% of the population who were known to have died but had no hospital activity in the last year of life for whom main exposure variables were lacking due to the lack of hospital record. This does not mean that all patients with no admissions were excluded, as a hospital record could be created without an inpatient admission. We have presented two sets of summary data for the ELSA participants, data for all deceased participants and data only for those with at least one hospital admission – neither are directly comparable to the Bardsley *et al* sample based on the exclusion criteria used.

The ELSA data reports admissions in the last 2 years of life, comparatively Bardsley *et al* report admissions in the last year of life. In Bardsley *et al*, 57% of last year of life admissions took place in the last 3 months of life, demonstrating the sharp increase in admissions closer to death. This suggests that the majority of admissions in the ELSA data are likely to have taken place in the last year of life, yet comparability between the ELSA data and the data reported in Bardsley *et al* is limited.

	Deceased ELSA participants (n=962*)	Deceased ELSA participants, excluding those with no hospital admissions (n=767)	All decedents in England 2009-2012, excluding those with no hospital record (n=1,223,859)
Mean (sd)	2.09 (3.65)	2.62 (3.91)	2.28 (2.17)
Median (10th centile-90th centile)	1.00 (0.00-4.00)	2.00 (1.00-5.00)	2.00 (0.00-5.00)

*excluding 14 participants with missing hospital admission information

2: Technical information on Mplus analysis

Standardisation procedure

Coefficients were standardised in Mplus using STDYX standardisation for continuous covariates, interpreted as 'the change in y in y standard deviation units for a standard deviation change in x', and STDY standardisation for the binary gender variable, interpreted as 'the change in y in y standard deviation units when x changes from zero to one'.[3] In both standardisation approaches y is y* (the continuous latent response variable underlying the binary variable y).

Confirmatory Factor Analysis (CFA)

For the CFA, we used the default robust weighted least squares (WLSMV) estimator in Mplus, generating linear regression coefficients for continuous items and probit regression coefficients for binary and categorical items.[3] Models were estimated using all available data.[4]

Preliminary analysis

In the preliminary analysis when analysing paths between exposures, mediators and outcomes in separate regression models, we used linear regression and maximum likelihood (ML) estimator for continuous outcomes, and probit regression with (WLSMV) estimator for binary outcomes. For the preliminary single mediator models we used the WLSMV estimator and coefficients were probits.

Full structural model

We used the WLSMV estimator available as default in Mplus for binary outcomes.[5] WLSMV is computationally more efficient than ML and has the advantage of producing absolute model fit statistics.[5] As a result of using WLSMV our coefficients are based on probit regression (logit models available with ML are not available within the more general WLSMV framework). Logit and probit models are both generalised linear models appropriate for binary outcomes, the former uses the logit link function, the latter the inverse normal link function.[6] In practice probit and logit models have similar fit to data.[6] The parameter estimates from logistic regression are approximately 1.8 times those from probit models.[6] Unlike logits, probits cannot be exponentiated to an odds ratio. We translated some of the unstandardised probit coefficients to probabilities for the dependent variable at different values of the independent variable of interest, using the standard formula[3, 7]:

$$P(u_i = 1|x_i) = F(\beta_0 + \beta_1 x_i)$$

Where F is the standard normal distribution, we looked up the result from $(\beta_0 + \beta_1 x_i)$ on a z-score table to get the probability. When calculating the probabilities we centered all covariates so their means were zero, thus probabilities are interpreted as those for a man with average scores on all other numerical covariates.

Missing data

The default in Mplus is to use all available information, rather than listwise deletion. With ML a Full Information Maximum Likelihood (FIML) approach is used, this method is robust with missing at random (MAR) data patterns where systematic missingness is accounted for by covariates included in the model. With WLSMV, missing data assumptions are more restrictive and limited to missing at random with respect to independent variables (MARX), i.e estimates are not robust if missingness is affected by any dependent variables in the model.[4]

The proportion of missing data was low (<5%) for all variables apart from the latent social support variable (26.1% missing). The missing data on latent social support was reasonably assumed to be MAR but was associated with both independent and dependent variables in our model and therefore violated the MARX assumption. To address missing data in the social support variable the multiple imputation (MI) feature in Mplus was used.

Multiple imputation (MI)

MI in Mplus uses Bayesian estimation to create multiple copies of the datasets with missing values imputed from the predictive distribution based on the observed data. Parameter estimates are then averaged over the set of datasets and standard errors computed from the average of the squared standard errors across the set and from the between analysis parameter estimate variation.[3] In our study, missing values were predicted by all variables included in the final model and 30 imputed datasets were created (representative of the proportion of missing data on the social support variable).

3: Descriptive analysis of the latent variables

Health and function

Items underlying health and function at final wave	N=737<br>Mean (sd)/n (%)
Grip strength (kg), mean (sd)	25.8 (10.2)
missing, n (%)	538 (73.0)
FVC (litres), mean (sd)	2.6 (0.9)
missing, n (%)	584 (79.2)
5 chair rises (seconds), mean (sd)	49.4 (6.8)
missing, n (%)	616 (83.6)
≥1 functional limitation*, n (%)	434 (58.9)
No functional limitation, n (%)	285 (38.7)
missing, n (%)	18 (2.4)
≥1 chronic illness*, n (%)	542 (73.5)
No chronic illness, n (%)	194 (26.3)
missing, n (%)	1 (0.1)
Poor self-rated health, n (%):	
Poor	116 (15.7)
Fair	125 (17.0)
Good	120 (16.3)
Very good or excellent	72 (9.8)
missing, n (%)	304 (41.3)

*functional limitations include: walking 100 yards (n=284), climbing a single flight of stairs (n=275), and carrying >10lbs (n=373); chronic illnesses include: chronic lung disease (n=133), asthma (n=103), arthritis (n=321), osteoporosis (n=74), cancer (n=166), parkinsons (n=9), any emotional or psychiatric illness (n=60), alzheimers (n=5), and dementia (n=23).

Access to healthcare services

Items underlying access to healthcare services at final wave	N=737<br>Mean (sd)/n (%)
Ease of access to GP, n (%):	
Difficult or unable	75 (10.2)
Quite easy	137 (18.6)
Very easy	207 (28.1)
missing, n (%)	318 (43.2)
Ease of access to Dentist, n (%):	
Difficult or unable	78 (10.6)
Quite easy	120 (16.3)
Very easy	150 (20.4)
missing, n (%)	389 (52.8)
Ease of access to Hospital, n (%):	
Difficult or unable	104 (14.1)
Quite easy	168 (22.8)
Very easy	145 (19.7)
missing, n (%)	320 (43.4)
Ease of access to Optician, n (%):	
Difficult or unable	86 (11.7)
Quite easy	149 (20.2)
Very easy	171 (23.2)
missing, n (%)	331 (44.9)
Transport deprived*, n (%)	105 (14.3)
Not transport deprived, n (%)	594 (80.6)
missing, n (%)	38 (5.2)
Unmet social care need*, n (%)	204 (27.7)
No unmet social care need, n (%)	596 (67.3)
missing, n (%)	37 (5.0)

* transport deprivation defined as: no access to car and limited access to public transport; unmet social care need defined as: an activity of daily living or instrumental activity of daily living need not being met by either social service, national health service or privately paid workers.

Social support

Following previous examples[8, 9] we used a series of self-reported items capturing the quality of relationships with children, family and friends. Three questions were asked about positive support: 'How much do they really understand the way you feel about things?'; 'How much can you rely on them if you have a serious problem?'; 'How much can you open up to them if you need to talk about your worries?' and three questions about negative support: 'How much do they criticise you?'; 'How much do they let you down when you are counting on them?'; 'How much do they get on your nerves?', each asked in relation to children, family and friends. Response categories were assigned a score of three for the most positive response, to zero for the least positive; those reporting having no children, family or friends were assigned a zero.

**Items underlying social support at final wave, n=737**		**0 n (%)**	**1 n (%)**	**2 n (%)**	**3 n (%)**	**missing n (%)**
1	how much your children understand you?	71 (9.6)	48 (6.5)	160 (21.7)	238 (32.3)	220 (29.9)
2	how much you can rely on your children?	67 (9.1)	24 (3.3)	52 (7.1)	383 (52)	211 (28.6)
3	how much you can open up to your children?	78 (10.6)	62 (8.4)	115 (15.6)	267 (36.2)	215 (29.2)
4	how much your family understand you?	113 (15.3)	116 (15.7)	141 (19.1)	142 (19.3)	225 (30.5)
5	how much you can rely on your family?	117 (15.9)	90 (12.2)	76 (10.3)	233 (31.6)	221 (30)
6	how much you can open up to your family ?	146 (19.8)	93 (12.6)	115 (15.6)	159 (21.6)	224 (30.4)
7	how much your friends understand you?	64 (8.7)	81 (11)	209 (28.4)	159 (21.6)	224 (30.4)
8	how much you can rely on your friends?	68 (9.2)	80 (10.9)	140 (19)	226 (30.7)	223 (30.3)
9	how much you can open up to your friends?	84 (11.4)	99 (13.4)	161 (21.8)	163 (22.1)	230 (31.2)
10	how much your children criticise you?	76 (10.3)	91 (12.3)	174 (23.6)	175 (23.7)	221 (30)
11	how much your children let you down?	72 (9.8)	33 (4.5)	78 (10.6)	335 (45.5)	219 (29.7)
12	how much your children get on your nerves?	68 (9.2)	40 (5.4)	141 (19.1)	272 (36.9)	216 (29.3)
13	how much your family criticise you?	74 (10)	67 (9.1)	136 (18.5)	220 (29.9)	240 (32.6)
14	how much your family let you down?	89 (12.1)	45 (6.1)	82 (11.1)	289 (39.2)	232 (31.5)
15	how much your family get on your nerves?	76 (10.3)	31 (4.2)	144 (19.5)	254 (34.5)	232 (31.5)
16	how much your friends criticise you?	49 (6.6)	51 (6.9)	136 (18.5)	267 (36.2)	234 (31.8)
17	how much your friends let you down?	54 (7.3)	38 (5.2)	81 (11)	333 (45.2)	231 (31.3)
18	how much your friends get on your nerves?	54 (7.3)	27 (3.7)	129 (17.5)	300 (40.7)	227 (30.8)

Higher scores are more optimal. Responses for positive items (1-9) are: 0 a lot, 1 some, 2 a little, 3 not at all, for the negative items (10-18) options are reversed.

Summary of latent variable measurement models

	**Health and function**	**Access to healthcare services**	**Social support**
Model fit:<br>Chi square<br><br>RMSEA<br>CFI<br>TLI	 $\chi^2(8) = 18.503$<br>(p = 0.0178)<br>0.042<br>0.974<br>0.952	 $\chi^2(9) = 11.485$<br>(p = 0.2439)<br>0.020<br>1.000<br>1.000	 χ^2 (123) = 437.453<br>(p <0.001)<br>0.068<br>0.985<br>0.982
N (cases with non-missing factor score)	737	711	545
Factor score<br>Mean (sd)<br>Median (interquartile range)	 .00 (.12)<br>-.01 (-.09, .11)	 -.08 (.66)<br>-.06 (-.43, .30)	 -.04 (.28)<br>-.03 (-.18, .12)

Standardised factor loadings

**Items**	**Health and function**	**Access to healthcare services**	**Social support**
Grip strength	0.179		
FVC	0.319		
Chair rise	0.263		
functional limitation	0.844		
Chronic illness	0.585		
Self-rated health	0.744		
Access to GP		0.944	
Access to dentist		0.962	
Access to optician		0.953	
Access to secondary care		0.847	
Unmet social care need		0.521	
Transport deprivation		0.600	
Children support			0.469
Family support			0.720
Friends support			0.497

4: Preliminary analysis

We analysed paths between exposures, mediators and outcomes in separate regression models, adjusted for age and gender. Wealth and education effects were mutually adjusted for each other (but not in a sequential relationship as they were in the final model). Fixed factor scores were used and data was non-imputed, all available cases were analysed. Standardised effects are reported.

Standardised effects for relationships between exposures, mediators and outcomes

Relationship	n	STDYX effect
wealth > health	731	**0.24 (0.12, 0.32)**
education > health	731	0.00 (-0.08, 0.08)
wealth > access	707	**0.19 (0.12, 0.27)**
education > access	707	0.03 (-0.05, 0.11)
wealth > social	542	0.05 (-0.04, 0.14)
education > social	542	0.00 (-0.09, 0.09)
wealth > place of death	731	**-0.15 (-0.25, -0.06)**
education > place of death	731	-0.09 (-0.18, 0.01)
health > place of death	737	-0.07 (-0.15, 0.02)
access > place of death	711	0.00 (-0.09, 0.09
social > place of death	545	0.02 (-0.08, 0.13)
wealth > hospital admissions	720	**-0.12 (-0.23, -0.02)**
education > hospital admissions	720	0.01 (-0.10, 0.12)
health > hospital admissions	726	**-0.19 (-0.29, -0.09)**
access > hospital admissions	700	-0.10 (-0.20, 0.01)
social > hospital admissions	539	-0.06 (-0.17, 0.05)

> indicates the direction of the regression path, i.e. wealth predicts health.

We then modelled each of the mediators in a separate structural model that included the two outcomes simultaneously, education and wealth (with wealth regressed on education in a sequential relationship), and adjusted for the effects of age and gender on the mediator and outcomes. Data was non-imputed, all available cases were analysed.

Standardised direct and indirect effects for wealth and education on death in hospital and hospital admissions via health and function, access to healthcare services, and social support: single mediator models

	**n**	**Death in hospital**	**≥3 hospital admissions**
**Model 1: health and function**	736		
Wealth (direct effect)		**-0.15 (-0.24, -0.05)**	-0.08 (-0.19, 0.02)
Indirect effect via health and function		-0.01 (-0.03, 0.02)	**-0.04 (-0.07, -0.01)**
Education (direct effect)		-0.08 (-0.18, 0.01)	0.01 (-0.10, 0.12)
Indirect effect via health and function		0.00 (-0.00, 0.00)	-0.00 (-0.01, 0.01)
**Model 2: access to healthcare services**	736		
Wealth (direct effect)		**-0.16 (-0.26, -0.07)**	**-0.11 (-0.21, -0.00)**
Indirect affect via access to healthcare services		0.01 (-0.01, 0.03)	-0.01 (-0.04, 0.01)
Education (direct effect)		-0.09 (-0.18, 0.01)	0.01 (-0.10, 0.12)
Indirect affect via access to healthcare services		0.00 (-0.00, 0.01)	-0.00 (-0.01, 0.01)
**Model 3: social support**	736		
Wealth (direct effect)		**-0.15 (-0.25, -0.06)**	**-0.12 (-0.22, -0.02)**
Indirect effect via social support		0.00 (-0.00, 0.00)	-0.00 (-0.01, 0.01)
Education (direct effect)		-0.08 (-0.18, 0.01)	0.01 (-0.10, 0.12)
Indirect effect via social support		0.00 (-0.00, 0.00)	0.00 (-0.01, 0.01)

5: Interaction effect showing the direct effect (and 95% CI) of wealth on health and function moderated by age

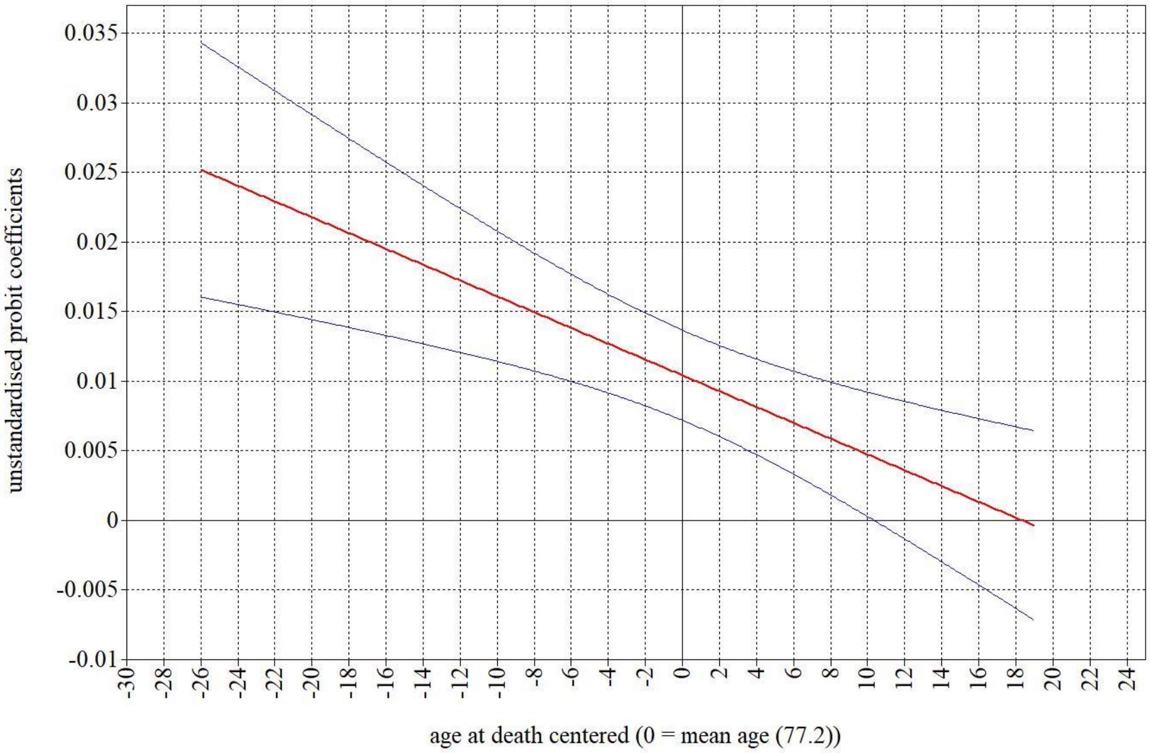

6: Standardised probit coefficients with bias corrected bootstrap confidence intervals for the direct and indirect effects for the final structural model (non-imputed data), n=736

	**wealth**	**health and function**	**access to healthcare services**	**social support**	**death in hospital**	**≥3 hospital admissions**
**Covariates**						
Age	-	-0.05 (-0.10, 0.02)	**-0.23 (-0.28, 0.17)**	**0.12 (0.05, 0.19)**	**0.26 (0.19, 0.34)**	**-0.14 (-0.23, -0.05)**
Female gender		-0.12 (-0.26, 0.03)	0.10 (-0.03, 0.23)	**0.28 (0.11, 0.43)**	-0.01 (-0.20, 0.17)	-0.15 (-0.34, 0.06)
**Mediators**						
Health and function (direct effect)	-	-	**0.38 (0.33, 0.43)**	-	-0.05 (-0.13, 0.04)	**-0.17 (-0.26, -0.08)**
Indirect effect via access to healthcare services	-	-	-	-	0.02 (-0.01, 0.06)	0.00 (-0.04, 0.04)
Access to healthcare services (direct effect)	-	-	-	-	0.06 (-0.02, 0.15)	0.00 (-0.09, 0.09)
Social support (direct effect)	-	-	-	-	0.03 (-0.06, 0.12)	-0.05 (-0.15, 0.05)
**Wealth**						
Direct effect	-	**0.25 (0.19, 0.32)**	**0.10 (0.04, 0.16)**	0.07 (-0.01, 0.15)	**-0.16 (-0.24, -0.07)**	-0.08 (-0.17, 0.01)
Total indirect effects	-	-	-	-	0.00 (-0.02, 0.03)	**-0.05 (-0.08, 0.02)**
Via health and function	-	-	**0.10 (0.07, 0.13)**	-	-0.01 (-0.03, 0.01)	**-0.04 (-0.07, -0.02)**
Via access to health-care services	-	-	-	-	0.01 (-0.00, 0.02)	0.00 (-0.01, 0.01)
Via social support	-	-	-	-	0.00 (-0.00, 0.01)	-0.00 (-0.02, 0.00)
Via health and function, and access to health-care services	-	-	-	-	0.01 (-0.00, 0.02)	0.00 (-0.01, 0.01)
Total effect	-	-	-	-	**-0.15 (-0.23, -0.07)**	**-0.12 (-0.21, -0.03)**
**Highest educational qualification**						
Direct effect	**0.39 (0.33, 0.44)**	0.00 (-0.06, 0.07)	0.03 (-0.03, 0.09)	-0.00 (-0.08, 0.07)	**-0.09 (-0.17, -0.00)**	0.00 (-0.08, 0.10)
Total indirect effects	-	-	-	-	**-0.06 (-0.09, -0.03)**	**-0.05 (-0.09, -0.01)**
Via health and function	-	-	0.00 (-0.03, 0.03)	-	-0.00 (-0.01, 0.00)	0.00 (-0.01, 0.01)
Via access to health-care services	-	-	-	-	0.00 (-0.00, 0.01)	0.00 (-0.00, 0.00)
Via social support	-	-	-	-	0.00 (-0.01, 0.00)	0.00 (-0.00, 0.01)
Via wealth	-	**0.10 (0.07, 0.13)**	**0.04 (0.02, 0.06)**	0.03 (-0.00, 0.06)	**-0.06 (-0.10, -0.03)**	-0.03 (-0.07, 0.01)
Via health and function, and access to health-care services	-	-	-	-	0.00 (-0.00, 0.00)	0.00 (-0.00, 0.00)
Total effect	-	-	-	-	**-0.14 (-0.22, -0.07)**	-0.04 (-0.12, 0.04)
**Covariances**						
Social support	-	-	**0.11 (0.04, 0.19)**	-	-	**-**
Death in hospital	-	-	-	-	-	**0.21 (0.10, 0.32)**

Effects with a p value <0.05 are shown in bold.

Model fit: $\chi^2(3) = 5.062$ (p = 0.1673), RMSEA = 0.031 CFI = 0.994, TLI = 0.933

7: Standardised probit coefficients for the direct and indirect effects for the full structural model, n=950 including participants with an admission to a care home in the last 2 years of life

	**Wealth**	**Health and function**	**Access to healthcare services**	**Social support**	**Death in hospital**	**≥3 hospital admissions**
**Covariates**						
Age	-	**-0.10 (-0.17, -0.04)**	**-0.24 (-0.29, -0.18)**	**0.12 (0.04, 0.19)**	**0.13 (0.05, 0.22)**	**-0.13 (-0.22, -0.04)**
Female gender		**-0.25 (-0.37, -0.12)**	0.07 (-0.05, 0.19)	**0.27 (0.12, 0.42)**	-0.06 (-0.22, 0.11)	-0.12 (-0.30, 0.06)
**Mediators**						
Health and function (direct effect)	-	-	**0.38 (0.32, 0.43)**	-	-0.04 (-0.13, 0.05)	**-0.15 (-0.25, -0.05)**
Indirect effect via access to healthcare services	-	-	-	-	0.04 (-0.00, 0.07)	-0.00 (-0.04, 0.04)
Access to healthcare services (direct effect)	-	-	-	-	0.09 (-0.00, 0.19)	-0.01 (-0.11, 0.09)
Social support (direct effect)	-	-	-	-	0.01 (-0.92, 0.11)	-0.02 (-0.12, 0.08)
**Wealth**						
Direct effect	-	**0.23 (0.17, 0.30)**	**0.08 (0.01, 0.14)**	0.06 (-0.03, 0.16)	**-0.09 (-0.18, -0.01)**	-0.09 (-0.18, 0.01)
Total indirect effects	-	-	**-**		0.01 (-0.02, 0.03)	**-0.04 (-0.06, -0.01)**
Via health and function	-	-	**0.09 (0.06, 0.12)**	-	-0.01 (-0.03, 0.01)	**-0.04 (-0.06, -0.01)**
Via access to health-care services	-	-	-	-	0.01 (-0.00, 0.02)	-0.00 (-0.01, 0.01)
Via social support	-	-	-	-	0.00 (-0.01, 0.01)	-0.00 (-0.01, 0.01)
Via health and function, and access to health-care services	-	-	-	-	0.01 (-0.00, 0.02)	-0.00 (-0.01, 0.01)
Total effect	-	-	-	-	**-0.09 (-0.17, -0.00)**	**-0.13 (-0.22, -0.03)**
**Highest educational qualification**						
Direct effect	**0.39 (0.34, 0.45)**	0.01 (-0.06, 0.08)	0.01 (-0.05, 0.08)	0.01 (-0.08, 0.09)	-0.09 (-0.18, 0.00)	0.04 (-0.06, 0.13)
Total indirect effects	-	-	-	-	-0.03 (-0.07, 0.00)	**-0.05 (-0.09, -0.01)**
Via health and function	-	-	0.00 (-0.02, 0.03)	-	0.00 (-0.00, 0.00)	-0.00 (-0.01, 0.01)
Via access to health-care services	-	-	-	-	0.00 (-0.01, 0.01)	0.00 (-0.00, 0.00)
Via social support	-	-	-	-	0.00 (-0.00, 0.00)	0.00 (-0.00, 0.00)
Via wealth	-	**0.09 (0.06, 0.12)**	**0.03 (0.00, 0.06)**	0.03 (-0.1, 0.06)	**-0.04 (-0.07, -0.00)**	-0.04 (-0.01, 0.00)
Via health and function, and access to health-care services	-	-	-	-	0.00 (-0.00, 0.00)	0.00 (-0.00, 0.00)
Total effect	-	-	-	-	**-0.12 (-0.20, -0.04)**	-0.02 (-0.10, 0.08)
**Covariances**						
Social support	-	-	**0.15 (0.07, 0.22)**	-	-	**-**
Death in hospital	-	-	-	-	-	**0.22 (0.11, 0.32)**

Effects with a p value <0.05 are shown in bold.

(model fit indices were averaged across the MI sets): $\chi^2(3) = 11.201$ (sd = 2.857), RMSEA = 0.053 (sd = 0.009) and CFI = 0.982 (sd = 0.006) indicate good fit, and TLI = 0.802 (sd = 0.068) indicates less than adequate fit.

8: Sensitivity analysis: cause of death and depressive symptoms as potential confounders

To investigate diagnosis as a confounder of the exposure-outcome relationships, we describe the distribution of exposures, mediators and outcomes by cause of death and diagnosis of depressive symptoms. We also present the direct effect of wealth on the outcomes for two additional models, one with the effects on the outcomes adjusted for cancer versus non-cancer cause of death, and another adjusted for diagnosis of depressive symptoms.

In this sample, the proportion of people who died in hospital (compared to home or hospice) is higher for people with a non-cancer cause of death and for people with depressive symptoms (tables 8a and 8b). Education and wealth are lower for people with a non-cancer cause of death and for people with depressive symptoms (tables 8a and 8b).

The direct effect of wealth on hospital death remains statistically significant but is attenuated when the final model is adjusted for cancer cause of death, and when the final model is adjusted for depressive symptoms (table 8c). The effect of wealth on hospital admissions remains statistically non-significant in the adjusted models.

[Scale of measures for table 8a and 8b (non-imputed data): educational qualifications (edqual) 0 to 5; wealth 1-10; health and function (health) -0.25 to 0.29; access to healthcare services (access) -1.52 to 0.89; social support (social) -0.86 to 0.59. Health, access and social are latent factor scores.]

8a Distribution of exposures, mediators and outcomes by cause of death*

Cause of death	N	Age, mean (SD)	edqual, mean (SD)	wealth, mean (SD)	health, mean (SD)	access, mean (SD)	social, mean (SD)	% death in hospital	% ≥3 hospital admissions
Cancer	251	73.7 (9.5) (n=251)	2.2 (1.5) (n=251)	5.7 (2.9) (n=250)	0.0 (0.1) (n=251)	0.1 (0.6) (n=244)	-0.0 (0.3) (n=200)	111 (44.2%)	70 (27.9%)
Cardiovascular	215	79.5 (9.1) (n=215)	1.7 (1.2) (n=215)	4.3 (2.8) (n=212)	-0.0 (0.1) (n=215)	-0.2 (0.6) (n=208)	-0.0 (0.3) (n=156)	138 (64.2%)	38 (17.7%)
Respiratory	92	79.2 (9.7) (n=92)	1.7 (1.2) (n=92)	4.5 (2.7) (n=91)	-0.0 (0.1) (n=92)	-0.3 (0.7) (n=91)	-0.1 (0.3) (n=66)	69 (75.0%)	33 (35.9%)
Other	84	77.9 (10.2) (n=84)	1.6 (1.1) (n=84)	4.9 (2.9) (n=84)	0.0 (0.1) (n=84)	-0.1 (0.7) (n=80)	0.0 (0.3) (n=62)	68 (81.0%)	18 (21.4%)
missing	95	78.9 (9.9) (n=95)	1.9 (1.3) (n=94)	5.5 (2.9) (n=95)	0.0 (0.1) (n=95)	-0.1 (0.6) (n=88)	-0.1 (0.3) (n=61)	63 (66.3%)	28 (29.5%)

*cause of death from death record

8b Distribution of exposures, mediators and outcomes by self-reported depressive symptoms*

	N	age, mean (SD)	edqual, mean (SD)	wealth, mean (SD)	somatic, mean (SD)	access, mean (SD)	social, mean (SD)	% death in hospital	% ≥3 hospital admissions
Depressive symptoms*	481	76.0 (9.8) (n=180)	1.7 (1.2) (n=481)	4.7 (2.8) (n=480)	-0.0 (0.1) (n=481)	-0.1 (0.7) (n=481)	-0.1 (0.3) (n=385)	308 (64.0%)	128 (26.6%)
No depressive symptoms	180	77.2 (9.8) (n=481)	2.3 (1.5) (n=180)	6.1 (2.8) (n=178)	0.1 (0.1) (n=180)	0.2 (0.6) (n=180)	0.0 (0.3) (n=152)	89 (49.4%)	41 (22.8%)
Missing	76	80.5 (9.8) (n=76)	1.8 (1.3) (n=75)	4.9 (2.9) (n=74)	-0.0 (0.1) (n=76)	-0.3 (0.5) (n=50)	-0.1 (0.3) (n=8)	52 (68.4%)	18 (23.7%)

*: ≥1 of the following self-reported depressive symptoms: depressed much of the time, everything an effort much of the time, sleep was restless much of the time, not happy much of the time, felt lonely much of the time, did not enjoy life much of the time, felt sad much of the time, could not get going much of the time

8c Direct effect of wealth on the outcomes after adjusting for cancer cause of death (versus non-cancer cause of death), and diagnosis of depressive symptoms (versus absence of depressive symptoms) (n=737)

	**Direct effect of wealth on death in hospital**	**Direct effect of wealth on hospital admissions**
**Final model** (not adjusted for cancer cause of death or diagnosis of depressive symptoms)	-0.16 (-0.25, -0.06)	-0.08 (-0.19, 0.03)
**Adjustment for cancer* (0=non-cancer cause of death; 1=cancer cause of death)**	-0.12 (-0.22, -0.03)	-0.09 (-0.20, 0.01)
**Adjustment for depressive symptoms** (0=no depressive symptoms; 1= ≥1 depressive symptoms)**	-0.14 (-0.24, -0.05)	-0.09 (-0.19, 0.02)

***model fit following adjustment for cancer:** $\chi^2(7) = 47.587$ (sd = 6.035), RMSEA = 0.088 (sd =0.007) and CFI = 0.891 (sd = 0.015), TLI = 0.393 (sd =0.086).

****model fit following adjustment for depressive symptoms:** $\chi^2(3) = 138.773$ (sd = 6.577), RMSEA = 0.160 (sd =0.004) and CFI = 0.704 (sd = 0.014), TLI = -0.649 (sd = 0.076).

References

1. Gao W, Ho Y, Verne J, et al. Geographical and temporal Understanding In place of Death in England (1984–2010): analysis of trends and associated factors to improve end-of-life Care (GUIDE_Care) – primary research. Health Serv Deliv Res. 2014;2(42).
2. Bardsley M, Georghiou T, Spence R, et al. Factors associated with variation in hospital use at the end of life in England. BMJ Supportive & Palliative Care. 2019;9(2):167-174.
3. Muthén L, Muthén B. Mplus User’s Guide. Sixth Edition. Los Angeles, CA: Muthén & Muthén 1998-2010.
4. Asparouhov T, Muthén, BO. Weighted Least Squares Estimation with Missing Data. unpublished article, available: http://wwwstatmodelcom/download/GstrucMissingRevisionpdf. (accessed: 16.02.2020).
5. Muthén B, Muthén., L. & Asparouhov, T. Estimator choices with categorical outcomes. unpublished technical report, available: http://wwwstatmodelcom/download/EstimatorChoicespdf. 2015;(accessed: 10.03.2020).
6. Agresti A. An introduction to categorical data analysis: second edition. Hoboken, New Jersey: John Wiley & Sons, Inc. 2007.
7. Muthén BO, Muthén LK, Asparouhov T. Regression and Mediation Analysis Using Mplus. Los Angeles, CA: Muthen & Muthen 2016.
8. Banks J, Berkman L, Smith J, et al. Do cross-country variations in social integration and social interactions explain differences in life expectancy in industrialized countries? . In Crimmins E, Preston S, Cohen B, (Eds). International differences in mortality at old ages: dimensions and sources. Washington, DC: The National Academies Press 2010.
9. Ding YY, Kuha J, Murphy M. Multidimensional predictors of physical frailty in older people: identifying how and for whom they exert their effects. Biogerontology. 2017;18(2):237-252.
